# Octa-repeat domain of the mammalian prion protein mRNA forms stable A-helical hairpin structure rather than G-quadruplexes

**DOI:** 10.1038/s41598-019-39213-2

**Published:** 2019-02-21

**Authors:** Andreas Czech, Petr V. Konarev, Ingrid Goebel, Dmitri I. Svergun, Peter R. Wills, Zoya Ignatova

**Affiliations:** 1 Institute of Biochemistry and Molecular Biology University of Hamburg, Hamburg, Germany; 2 A. V. Shubnikov Institute of Crystallography of Federal Scientific Research Centre “Crystallography and Photonics” of Russian Academy of Sciences, Moscow, Russia; 3 National Research Centre “Kurchatov Institute”, Moscow, Russia; 4 European Molecular Biology Laboratory, Hamburg Outstation, c/o DESY, Hamburg, Germany; 5 Department of Physics, University of Auckland, Auckland, New Zealand

**Keywords:** RNA, SAXS

## Abstract

Misfolding and aggregation of prion protein (PrP) causes neurodegenerative diseases like Creutzfeldt-Jakob disease (CJD) and scrapie. Besides the consensus that spontaneous conversion of normal cellular PrP^C^ into misfolded and aggregating PrP^Sc^ is the central event in prion disease, an alternative hypothesis suggests the generation of pathological PrP^Sc^ by rare translational frameshifting events in the octa-repeat domain of the PrP mRNA. Ribosomal frameshifting most commonly relies on a slippery site and an adjacent stable RNA structure to stall translating ribosome. Hence, it is crucial to unravel the secondary structure of the octa-repeat domain of PrP mRNA. Each of the five octa-repeats contains a motif (GGCGGUGGUGGCUGGG) which alone *in vitro* forms a G-quadruplex. Since the propensity of mRNA to form secondary structure depends on the sequence context, we set to determine the structure of the complete octa-repeat region. We assessed the structure of full-length octa-repeat domain of PrP mRNA using dynamic light scattering (DLS), small angle X-ray scattering (SAXS), circular dichroism (CD) spectroscopy and selective 2′-hydroxyl acylation analysis by primer extension (SHAPE). Our data show that the PrP octa-repeat mRNA forms stable A-helical hairpins with no evidence of G-quadruplex structure even in the presence of G-quadruplex stabilizing agents.

## Introduction

Alternatively folded form of mammalian prion protein (PrP), broadly termed PrP^Sc^ (Sc = scrapie), is the molecular culprit of transmissible spongiform encephalopathies (TSE), fatal neurodegenerative diseases of infectious, hereditary, or spontaneous origin in susceptible hosts^1,2^. At the molecular level, misfolding and aggregation of PrP are associated with disease and its propagation. Consequently, seeding–induced aggregation serves as a sensitive assay of potential infectivity^3,4^. However, there is still debate about what initially triggers chain-reaction conformational changes in the PrP that dominates most infectious preparations^5^. Many studies convincingly suggest structural transition from α-helical to β-sheet structures in the C-terminal region of PrP^6^. Alternatively, other studies indicate the obligatory involvement of the N-terminal region^7–9^, e.g., amino acid residues 23–100 in human PrP, which is intrinsically disordered in aqueous solution^10^. The N-terminal region is capable of binding metal ions and polyanions, and following structural transitions it interacts with other cellular proteins, such as the neuronal cell adhesion molecule fibronectin domain^11^. In particular, the octa-repeat region (amino acid residues 51–91) within the N-terminal domain of PrP appears crucial for aggregation^12^. Interestingly, this region contains a variable number of octa-repeats both within individuals of any mammalian species and between the predominant phenotypes from any species. Most commonly, unaffected individual humans have four or five repeats but up to 13 repeats are associated with inherited disease, with an apparent dose-dependent effect in the penetrance and severity of hereditary TSE with increasing repeat number expansion^13,14^.

The discovery of a short –1 *trans*-frame product of the PrP gene termed “alternative PrP” (Alt-PrP) and spanning the octa-repeat region^15^, suggests the potential involvement of ribosomal frameshifting in the molecular processes whereby the infectious form of PrP is produced in cells^16^. Alt-PrP is formed when translation is initiated at an alternative AUG codon in the –1 reading frame at 90–92 bp in the human ORF and terminated at the first stop codon encountered in that frame (TAA at 309–311 bp). A slightly longer version of Alt-PrP could be produced as a result of translation initiation at the canonical start codon followed by ribosomal frameshifting at a suitable site in the octa-repeat region of the PrP ORF. Programmed –1 ribosomal frameshifting primarily described in viruses^17^ has also been reported in bacteria^18,19^, yeast^20^, as well as mice and humans^21,22^. Two elements embedded in the mRNA stimulate frameshifting: first, a slippery sequence, usually with the pattern X XXY YYZ, which is shifted to XXX YYY – both decoded by the same tRNAs through XX(Y/X) and YY(Z/Y) codon wobbling^23^; second, downstream of the slippery site, a stable RNA secondary structure, e.g., stem-loop^24,25^, pseudoknot^25,26^ or G-quadruplex^27,28^. Intriguingly, the octa-repeat region of PrP mRNA is known to have a rich potential to form stem-loop^29^, pseudoknot^30^ or G-quadruplex^31^ structures, all of which are associated with programmed frameshift events in other contexts^17^, but there is a dearth of experimental evidence which would verify any role for these structures^32^. Our interest was raised by the most recent study on PrP mRNA structure which suggested G-quadruplexes^31^, since it has been shown that G-quadruplexes in combination with an adjacent appropriate slippery site stimulate frameshifting^27,28^. Each of the five nearly identical 24-nt motifs in the human mRNA bears a potential unusual slippery site (C UGG GGG). Upon −1 frameshifting the A-site codon GGG could re-pair to the same tRNA, whereas the P-site codon would not re-pair. The −1 *trans*-frame product would be rich in hydrophobic amino acids, especially tryptophanes, potentially increasing the misfolding and aggregation propensity of such a PrP variant. In this study, we set out to determine the solution structure of the mRNA encoding the PrP octa-repeat region (94–288 nt of human ORF). Olsthoorn found that the PrP octa-repeat region has a propensity to form G-quadruplexes, but fairly short (24 nt) sequences, which form a G-quadruplex in the presence of KCl were used^31^. However, these structures compete with stem-loop formation in the presence of MgCl_2_^31^. Thus, we analyzed the whole 195 nt-long octa-repeat region. We hypothesized two alternative outcomes: 1) the accumulation of five G-quadruplexes within the octa-repeat domain would have a stacking effect^33,34^ leading to potential stabilization of the G-quadruplexes; or 2) the flanking sequences would outcompete the G-quadruplex formation, resulting in the complete octa-repeat domain taking up an alternative structure.

## Results

### The full-length PrP octa-repeat domain does not form G-quadruplexes

**Figure 1:**
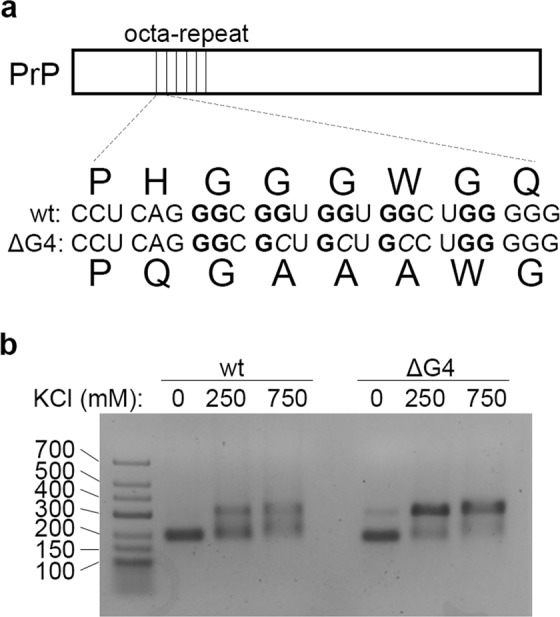
The octa-repeat region of the PrP RNA carries putative G-quadruplexes, which can be omitted by G-to-C substitutions. (**a**) Schematic representation of the introduced G-to-C-substitutions in the ΔG4 PrP octa-repeat mRNA to alter putative G-quadruplex motifs. (**b**) Native agarose gel electrophoresis shows oligomerization of wt and PrP octa-repeat mRNA.

Since short stretches of the PrP octa-repeat mRNA form G-quadruplexes^31^, we first addressed whether the complete octa-repeat mRNA can form such structures. A negative control incapable of forming G-quadruplexes (ΔG4, Fig. 1a) was generated in which cytosine residues were strategically substituted for guanine residues. The chosen mutations would also impair pseudoknot formation in the octa-repeat RNA, which had been proposed earlier^30^. Both wildtype (wt) and ΔG4 PrP octa-repeat stretches were transcribed *in vitro* and analyzed by native agarose gel electrophoresis. In the presence of various salts, including KCl, which is known to favor G-quadruplex formation (Fig. 1b), wt *and* ΔG4 PrP octa-repeat both formed oligomers (Supplementary Fig. 1). Thus, the oligomerization cannot be explained by the stacking of G-quadruplexes as shown for the telomeric TERRA RNA^33^. Instead, oligomerization of wt and ΔG4 PrP octa-repeat is likely to be the consequence of canonical Watson-Crick base pairing. As positive controls we analyzed known G-quadruplexes, e.g., the Epstein–Barr virus–encoded nuclear antigen 1 (EBNA1) which bears 12 two-layer (2 G) and one four-layer G-quadruplex (4 G) in its glycin-alanin repeat domain (GAr, Supplementary Fig. 2a)^35^. We treated the EBNA1 G-quadruplexes the same way as PrP mRNA and analyzed their oligomerization propensity by native gel electrophoresis. While the 2G G-quadruplex formed dimers and trimers in the presence of KCl, the 4G G-quadruplex assembled into large oligomers that did not enter the polyacrylamide gel (Supplementary Fig. 2b). In contrast, under the same conditions isolated stretches of the PrP mRNA, comprising the putative G-quadruplex solely (Pri-Qd) or additional nine nucleotides (Pri-One)^31^ failed to form high molecular weight oligomers (Supplementary Fig. 2c). Together, these data suggest that different G-quadruplexes form various oligomers, and the oligomerization of the PrP octa-repeat domain arises independently of G-quadruplex formation.

**Figure 2:**
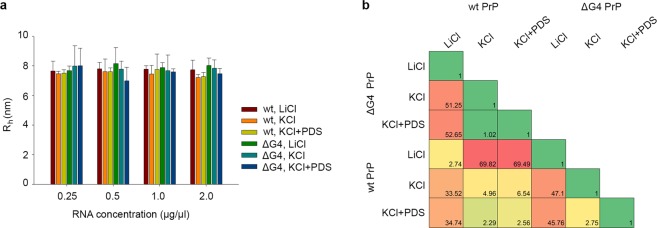
Both wt and ΔG4 PrP octa-repeat mRNA do not form G-quadruplex structures. (**a**) DLS reveals similar hydrodynamic radii (R_h_) of wt and ΔG4 PrP octa-repeat mRNA in different conditions. (**b**) SAXS measurements of wt and ΔG4 PrP octa-repeat mRNA in different conditions. The similarity was assessed by χ^2^ values calculated by the program DATCMP from ATSAS package^58^. Green and yellow denote moderate differences, red – significant difference.

Since G-quadruplex formation and stacking might occur only at higher concentrations and/or in the presence of additional G-quadruplex stabilizers, we used dynamic light scattering (DLS) to investigate the tertiary structure of PrP octa-repeat mRNA. DLS provides an estimate of the size of the molecules in pH-buffered aqueous solution. If the PrP octa-repeat mRNA could form G-quadruplexes, we expected different hydrodynamic radii (R_h_) in the presence of G-quadruplex-favoring KCl vs G-quadruplex-disfavoring LiCl. However, our results revealed no differences (Fig. 2a). Moreover, we also conducted measurements in the presence of pyridostatin (PDS), proved to be an effective G-quadruplex stabilizer in several studies^35–37^. We did not observe any differences in the estimated particle size of either wt or ΔG4 PrP octa-repeat RNA upon addition of KCl and PDS (Fig. 2a). Both wt and ΔG4 variant formed particles with similar radii of appr. 7.7 nm under all conditions (Fig. 2a), implying that none of them formed G-quadruplexes even under G-quadruplex-favoring conditions.

To gain insights into the size and shape of folded wt and ΔG4 PrP octa-repeat mRNA, we next performed SAXS measurements at different RNA concentrations and in the presence of 100 mM LiCl or KCl and 1 mM PDS. RNA preparation, SAXS measurements and data analysis were done using a procedure described previously for G-quadruplex dimers of an RNA aptamer^38^. The processed scattering data and the computed distance distribution functions are summarized in Supplementary Table S1 and Supplementary Fig. 3. The experimental radius of gyration (*R*_g_) and maximal distance (*D*_max_) of wild type form of PrP mRNA molecule in Tris buffer (7.35 ± 0.05 nm and 25.0 ± 1.0 nm, respectively) suggest a rather elongated structure. The *p*(*r*) function displayed an asymmetric tail (Supplementary Fig. 3b), typical for elongated particles. The ΔG4 mutation of PrP octa-repeat mRNA did not have a strong influence on these parameters whereas the addition of 100 mM KCl or LiCl to the buffer led to significant increases in *R*_g_ and *D*_max_ to 9.30 ± 0.07 nm and 31.0 ± 1.0 nm, respectively. These values correlated well with DLS measurements. The experimental molecular mass (*M*) of PrP octa-repeat mRNA in Tris buffer of 130 ± 20 kDa suggested that the RNA formed dimeric species in solution (theoretical *M* of the monomer is 72 kDa). The addition of KCl or LiCl promoted further oligomerization of PrP octa-repeat mRNA molecules, shifting the average *M* to 180 ± 20 kDa. This was corroborated by the excluded volume (*V*_p_) of the particle, 165 ± 20 nm^3^, which was in agreement with the empirical finding that the hydrated volume of compact RNA in nm^3^ is generally a factor of approximately 1.1 times larger than the *M* in kDa. Thus, in the concentration range of this study and under solvent conditions resembling the intracellular milieu the predominant species of PrP mRNA were dimers. However, the addition of KCl or LiCl caused the appearance of species of higher *M*, in corroboration with the salt-dependent oligomerization (Fig. 1). The differences between wt and ΔG4 PrP octa-repeat mRNA in buffers containing salts was also assessed from similarity matrix analyses (Fig. 2b) where χ^2^ values between different curves are reported. Significant differences were observed during transition from LiCl to KCl/KCl + PDS buffer, whereas the differences between wt and ΔG4 PrP octa-repeat mRNA in the same buffers were of one order of magnitude lower. This result suggests the absence of G-quadruplex under any of the conditions tested. The macromolecular shapes of individual PrP mRNA molecules have been reconstructed by *ab initio* modeling using the experimental X-ray scattering data. Low resolution models of the PrP mRNA particles in solution (Supplementary Fig. 4) were calculated using *ab initio* shape determination programs as described in Methods. They provided good fits to the experimental data with discrepancies in the range of χ^2^ = 1.13–1.54 (Supplementary Fig. 3a, solid lines and Supplementary Table 1). Individual molecules were found to resemble elongated, hairpin-like particles. Upon salt addition the fitted model shapes expanded in length and cross-section, but the overall topology remained the same. The ΔG4 PrP octa-repeat mRNA displayed more variability in its apparent shape, whereas the wt PrP octa-repeat mRNA was less dependent on the buffer type. In addition, comparison of DLS and SAXS results revealed that for all samples containing salts *R*_*h*_ < *R*_*g*_, which indicates rather flexible molecules^39^ (Fig. 2a, Supplementary Table 1). In summary, wt and ΔG4 PrP octa-repeat mRNA exhibited very similar size and shape under G-quadruplex-favoring and non-favoring conditions, arguing against G-quadruplex formation of this entire domain. One should note here that SAXS is very sensitive to the overall shapes and would allow to reliably detect G-quadruplexes if they were formed in solution^40^.

### PrP octa-repeat mRNA forms double-stranded A-helical structure

**Figure 3:**
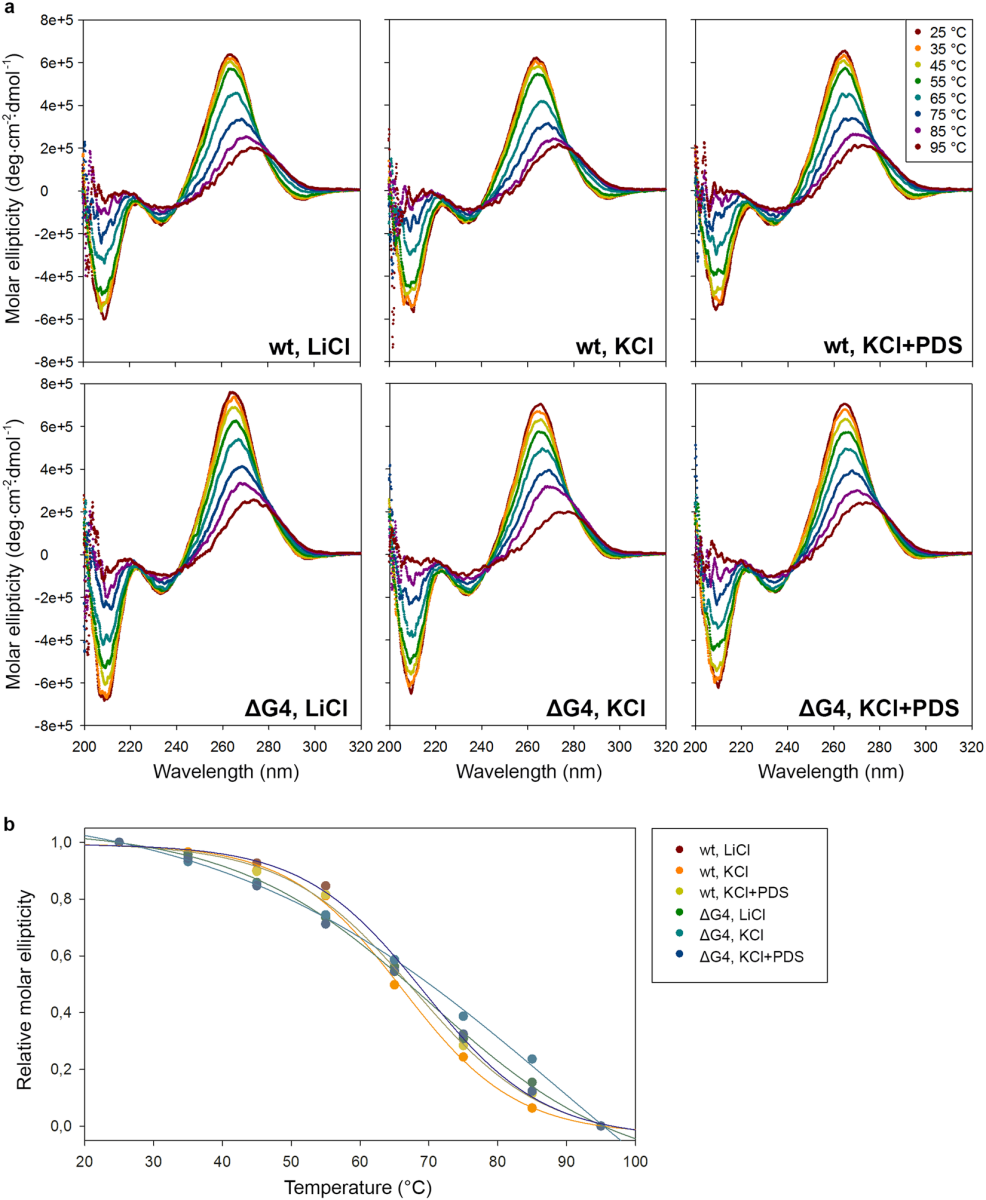
PrP octa-repeat mRNA adopts A-helical RNA structure. (**a**) CD spectra of wt and ΔG4 PrP octa-repeat mRNA under control (LiCl) and G-quadruplex-favouring conditions (KCl +/− PDS) at temperatures ranging from 25 to 95 °C show characteristic signature of A-helical dsRNA. (**b**) Melting curves were extracted from the maximal ellipticity of the prominent 264 nm peak and normalized to the molar ellipticity at 25 °C. Points were fitted to a 4 parameter sigmoidal curve.

Circular dichroism (CD) spectroscopy is commonly used to identify secondary structure motifs through their characteristic signatures in experimentally determined spectra. Therefore, we used CD spectroscopy to investigate the secondary structure of wt and ΔG4 PrP octa-repeat mRNA in the presence of LiCl, KCl only and KCl and PDS. Additionally, we conducted each measurement at different temperatures to calculate melting temperatures (*T*_*m*_). Both wt and ΔG4 PrP octa-repeat mRNA exhibited very similar spectra with a well-defined minimum at 210 nm, a weak minimum at 240 nm and a maximum at 264 nm (Fig. 3a). Such a spectral signature is characteristic of double-stranded, A-helical RNA^41^. Even in conditions stimulating the formation of G-quadruplex structure, i.e., KCl and PDS, we did not detect any G-quadruplex signature. Furthermore, the melting curves recorded at 264 nm and the melting temperatures were very similar between wt and ΔG4 PrP octa-repeat mRNA and did not depend on the salt in the solution (Fig. 3b). We conducted the same measurement with aforementioned isolated PrP fragments (Pri-One, Pri-Qd)^31^ and the 2 G and 4G G-quadruplexes from EBNA1^35^. In contrast to the full-length PrP RNA, these controls showed clearly different spectra when incubated with LiCl or KCl (Supplementary Fig. 5). First, the G-quadruplex-specific ellipticity at 264 nm increased in parallel with the appearance of the characteristic strong minimum at 240 nm (Supplementary Fig. 5a–d). Pri-One, forming A-helical hairpins without and G-quadruplexes with KCl, exhibited the hairpin-characteristic minimum at 210 nm in the presence of LiCl. This minimum disappeared following KCl incubation (Supplementary Fig. 5a), implying a structural shift from A-helical hairpin to G-quadruplex structure in isolated PrP fragments. In comparison, the minimum at 210 nm persisted in the complete PrP RNA even under conditions favoring G-quadruplex, e.g. with KCl and PDS (Fig. 3a). Moreover, the melting temperature increased in all four controls (Supplementary Fig. 5e,f), corroborating the formation of stable G-quadruplexes in the presence of KCl under our experimental conditions.

**Figure 4:**
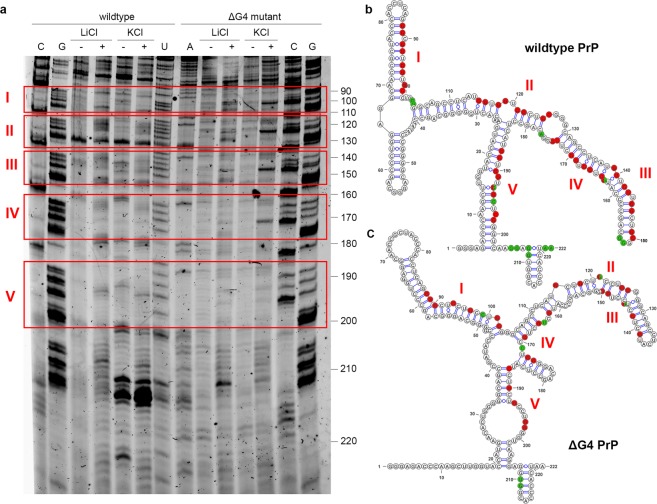
PrP octa-repeat mRNA is largely double-stranded. (**a**) SHAPE analysis of wt and ΔG4 PrP octa-repeat mRNA in 100 mM LiCl or KCl without (−) or with SHAPE reagent NMIA (+). The mRNA in the negative control (−) was incubated with DMSO. Red boxes indicate regions of putative G-quadruplex motifs. (**b**) RNA secondary structure representations from RNAfold. Single-stranded nucleotides from (a) are color coded in green. G’s crucial for a G-quadruplex formation are designated in red.

To determine further details of the secondary structure of the PrP octa-repeat mRNA, we employed SHAPE which can be used to identify single- and double-stranded RNA regions. The SHAPE reagent, NMIA, specifically modifies single-stranded RNA nucleotides, which leads to a termination of the subsequent reverse transcription and is detected on a 12.5% PAGE (Fig. 4a). Few bands were detected within the 90–200-nt stretch, suggesting that the region is engaged to a high degree in double-stranded structures. Moreover, we detected no significant difference in the susceptibility of PrP octa-repeat mRNA to NMIA following incubation with LiCl or KCl. At some positions the intensity of the bands from the NMIA-treated mRNA was higher than the control treated with DMSO. We used the RNAfold algorithm^42^ to predict the RNA secondary structure and found that both wt and ΔG4 PrP octa-repeat mRNA are likely to exhibit extended hairpin structures (Fig. 4b,c). Interestingly, the few single-stranded positions were very well matched to the SHAPE results (Fig. 4b,c; green nucleotides). Strikingly, guanines which could be engaged in a putative G-quadruplex (Fig. 4b; red nucleotides) are within single-stranded regions and were reactive to NMIA (G163, G194, Fig. 4b, green and red color-coded nucleotides). Also, regions expected to be base-paired in putative pseudoknots^30^ did not match with the bands in our SHAPE experiment. In contrast, in the isolated G-quadruplex-prone PrP fragment Pri-One, signals for guanine residues involved in G-qudruplex decreased or even completely vanished following KCl treatment (Supplementary Fig. 6a). Hence, while these residues are at least partially single-stranded and NMIA-reactive in LiCl buffer (Supplementary Fig. 6b), their NMIA-reactivity decreased when forming G-quartets stabilized by KCl (Supplementary Fig. 6c). Together, these results clearly support the notion that the full-length PrP octa-repeat mRNA adopts double-stranded A-helical structure, while isolated PrP fragments shift from A-helical hairpins to G-quadruplexes.

### The structure of PrP octa-repeat does not alter translation *in vivo*

**Figure 5:**
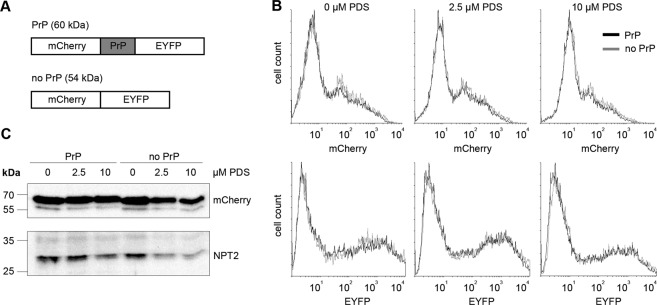
PrP octa-repeat domain does not alter expression in HeLa cells. (**A**) Reporter construct bearing the complete PrP octa-repeat domain sandwiched bewtween mCherry and EYFP. The construct with two fluorescent proteins only served as control. (**B**) Flow cytometry analysis of HeLa cells transfected with the reporter construct with or without PrP octa-repeat domain and treated with 0, 2.5 and 10 µM PDS (*n* = 2). (**C**) Representative immunoblot (*n* = 5) of full-length fusion protein detected by anti-mCherry antibody. NPT2 served as transfection control.

To address the impact of the wt PrP octa-repeat domain on translation *in vivo*, we cloned this region into a double-reporter plasmid sandwiched between two fluorescent proteins, mCherry and eYFP (Fig. 5A). We compared the expression of mCherry – PrP octa-repeat domain – eYFP in HeLa cells with a negative control comprising only the two fluorescent proteins, mCherry – eYFP using flow cytometry (Fig. 5B) and immunoblotting (Fig. 5C). The PrP insertion made no apparent difference in the efficiency of translation (Fig. 5B,C). To stabilize potential G-quadruplex structures in the PrP insert we exposed cells to different concentrations of PDS. Upon addition of PDS, we observed a global reduction of mCherry and eYFP expression which held true for constructs both with and without PrP insert. The effect increased with increasing PDS concentration. To minimize any effect that may have arisen from transfection efficiency, we added PDS 5–6 h after transfection and allowed cells to further synthesize the reporter for approx. 20 h but we still observed a general reduction in the expression level of both the constructs with increasing PDS concentration. On the other hand, this appeared to be a general PDS “background effect” that was of virtually the same magnitude whether or not the construct contained the PrP insert, indicating no effect in respect of the PrP octa-repeat domain forming G-quadruplexes or any other stable secondary structure *in vivo* which would repress mRNA translation (Supplementary Fig. 7d). Indeed, a qualitatively similar effect, but less marked, was observed in the detected level of translation of the NPT2 control gene (Supplementary Fig. 8B).To verify that our experimental conditions monitor repressing effects of G-quadruplexes on translation, we expressed the wildtype EBNA1 gene, bearing 13 G-quadruplexes in its GAr domain (Supplementary Fig. 2a) as well as a variant without this GAr domain (ΔGAr), both fused to GFP. The ΔGAr EBNA1 construct, missing the G-quadruplex motifs, showed a relative reduction of expression with increasing PDS concentration very similar to that previously observed with the mCherry/eYFP construct, either with or without the PrP insert (Supplementary Fig. 7b–d). In contrast, expression of wildtype EBNA1 was already reduced to a minimal level by 2.5 µM PDS and was observed at the same low level at 10 µM PDS, clearly demonstrating the repressing effect PDS-stabilized G-quadruplexes *in vivo* (Supplementary Fig. 7b–d). We noted that the general repressing effect of PDS was stronger for the PrP and ΔGAr EBNA1 constructs (Supplementary Fig. 8A) than for the control gene NPT2 (Supplementary Fig. 8B). This observation is explicable in terms of differential interaction of the drug with the cytomegalovirus (CMV) versus simian-virus 40 (SV40) promoter^43^. Alternatively, other potential G-quadruplexes within the sequences might be affected by PDS. According to QGRS mapper^44^, the NPT2 gene bears a lower number of potential G-quaduplexes, which have a lower average and lower maximal G-score compared with the other sequences (Supplementary Table 2). In summary, our investigation of the effect on translation of the G-quadruplex stabilizer PDS was complicated but not confounded by the drug’s general tendency to decrease the level of *in vivo* expression, even of the control gene NPT2, at least to some extent. Above this general tendency we showed a clear reduction of expression caused by the G-quadruplex-containing GAr domain in wt EBNA1 (Supplementary Fig. 7d). In contrast, in the PrP variants all of the effects of PDS could be accounted for in terms of its “background effect” (Supplementary Fig. 7d), corroborating our other data that failed to detect G-quadruplexes in the octa-repeat region of the PrP mRNA.

## Discussion

The octa-repeat domain of the prion protein mRNA contains five 24-nt motifs bearing potential G-quadruplex sequences (consensus: GGCGGUGGUGGCUGGG in human gene). In isolation this motif folds in the presence of KCl into a stable G-quadruplex (Supplementary Fig. 6c) with a melting temperature (*T*_*m*_) of 63 °C^31^. However, in the presence of MgCl_2_ the sequence forms a stem-loop RNA structure^31^. Since flanking regions play an important role and might compete with G-quadruplex formation, we studied the RNA structure of the entire 195 nt-long wt octa-repeat domain of human prion protein mRNA and compared it to a variant incapable of forming G-quadruplex (ΔG4). Using various biophysical and structural methods, our results did not reveal any G-quadruplex structure even in the presence of stabilizing KCl and PDS (Figs 1–4). Instead, we show that the PrP octa-repeat domain folds into stable A-helical stem-loop structure (Figs 3, 4). Several controls verified that G-quadruplexes can form under the chosen experimental conditions, including those previously observed in isolated fragments of PrP mRNA (Supplementary Figs 2, 5–7). The stability of G-quadruplexes depends on the number of stacked tetrades and intervening sequences^45,46^. The putative PrP G-quadruplexes bear one or two intervening nucleotides, but form only two stacked tetrads implying a rather low stability. On the other hand, consecutive G-quadruplexes can stack and stabilize each other^33^. Although our results show salt-dependent oligomerization of the PrP octa-repeat mRNA, this was independent of KCl and PDS and also occurred in the ΔG4 variant (Figs 1, 3). Similar two-stack G-quadruplexes are described for the mRNA of Epstein-Barr virus-encoded nuclear antigen 1 (EBNA1)^35^. Although the EBNA1 mRNA structure is less stable (*T*_*m*_ = 54 °C) than its PrP equivalent, the G-quadruplexes formed within the entire glycine-alanine repeat (GAr) domain (appr. 470 nt)^35^. The differential behavior of G-quadruplex-forming GAr RNA vis-à-vis stem-loop forming PrP octa-repeat RNA might be explained by the different nucleotide context. While both regions show similar GC content (67% vs. 71%), the amount of G and C is more balanced in PrP, allowing more GC base-pairing which may compete with the G-quadruplex formation. Additionally, the GAr domain bears 13 single G-quadruplex motifs (Supplementary Fig. 2a) which might stack and stabilize each other^35^. In the PrP octa-repeat domain there are only five, and stability gain from stacking might be too low to compete with stem-loop formation. In sum, flanking sequences of potential G-quadruplex motifs have a profound impact on the secondary structure and in the PrP octa-repeat mRNA stimulate stem-loop rather than G-quadruplex formation^47^.

Our results revealed no impact of the PrP octa-repeat domain on the expression of a fluorescent reporter gene. In contrast, the expression of G-quadruplex-rich EBNA-1 is repressed strongly by PDS. This is in agreement with recent publication which suggests a high propensity for sequences from the human genome to form G-quadruplexes *in vitro*, which are unstable *in vivo*^48^. Along this line, addition of PDS stabilized EBNA1 G-quadruplexes yielding a measurable reduction in expression, while under physiological conditions, i.e.,. 0 µM PDS, wildtype showed similar expression to the ΔGAr EBNA1 variant without G-quadruplexes (Supplementary Fig. 7c). The possibility, of stabilizing G-quadruplexes by intrinsic small molecules or proteins in the cell remains an interesting regulatory possibility. In contrast, since even *in vitro* full-length PrP mRNA does not form G-quadruplexes, it is highly unlikely that it will form G-quadruplexes *in vivo*.

## Material and Methods

### RNA preparation

The wildtype (wt) and mutant (G-to-C substitutions in the putative G-quadruplex motifs) PrP octa-repeat (bp 94–288 from the human PrP ORF) was cloned into pCDNA3 vector and *in vitro* transcribed by RiboMax Large RNA production kit (Promega). Plasmid was digested by DNase I treatment, RNA was purified by GeneJet RNA purification kit (Thermo Scientific) and integrity was checked on agarose gel. To assure proper folding, prior to any measurement, RNA was mixed with salts and/or pyridostatin (PDS, Sigma Aldrich) in the desired concentration, heated to 95 °C for 5 min and incubated at room temperature for at least 1 h.

### Circular dichroism (CD) spectroscopy

6 µM RNA in 10 mM Tris pH7.5 with 100 mM LiCl or KCl and 1 mM PDS was folded as described above and measured on a Jasco J-815 CD spectroscope by using a 10 mm quartz cuvette at 25 °C to 95 °C in 10 °C intervals. At each temperature, triplet spectra were accumulated over the spectral range from 200 to 320 nm at 0.1 nm intervals. Subsequently, the temperature was increased by 10 °C within 1 min and left at the new temperature for 2 min prior to commencing further data collection.

### Dynamic light scattering

0.25, 0.5, 1 and 2 µg/µl (3.4–27.5 µM) RNA in 10 mM Tris pH7.5 buffer with 100 mM LiCl or KCl and 1 mM PDS was folded as described above and measured on Spectro Size 300 (Xtal concepts) in a 10 mm quartz cuvette. The scattered autocorrelation function for each sample was determined twenty times by making measurements of 15 s duration. For each autocorrelation function, an estimate of the hydrodynamic radius of the molecules in the sample was calculated.

### Small angle x-ray scattering

0.25, 0.5, 1 and 2 µg/µl (3.4–27.5 µM) RNA in 10 mM Tris pH7.5 buffer with 100 mM LiCl or KCl and 1 mM PDS was folded as described above. Synchrotron radiation X-ray scattering data were collected on the EMBL P12 beamline on the storage ring PETRA III (DESY, Hamburg, Germany) at 20 °C^49^. The data were recorded using a 2 M PILATUS detector (DECTRIS, Switzerland) at a sample-detector distance of 3.0 m and a wavelength of *λ* = 0.124 nm, covering the range of momentum transfer 0.02 < *s* < 5.0 nm^–1^ (*s = *4*π* sin*θ*/*λ*, where 2*θ* is the scattering angle). No measurable radiation damage was detected by comparison of twenty successive time frames with 50 millisecond exposures. The data were averaged after normalization to the intensity of the transmitted beam, the scattering of the buffer was subtracted and the difference data were extrapolated to zero solute concentration using PRIMUS^50^.

The radius of gyration *R*_g_ of solute RNA molecule and the forward scattering *I*(0) were evaluated using the Guinier approximation at small angles (*s* < 1.3/*R*_g_)^51^ assuming the intensity was represented as *I*(*s*) = *I*(0) exp(−(*sR*_g_)^2^/3) and from the entire scattering pattern by the program GNOM^52^. In the latter case, the distance distribution function *p*(*r*) and the maximum particle dimension *D*_max_ were also computed. The molecular masses (*M*) of the molecules were evaluated by calibration against a reference solution of bovine serum albumin. The excluded volume of the hydrated molecule (*V*_p_) was calculated using the Porod approximation:

1 $${V}_{p}=2{\pi }^{2}I(0)/{\int }_{0}^{\infty }{s}^{2}{I}_{exp}(s)ds$$

in which the intensity *I*(*s*) was modified by subtraction of an appropriate constant from each data point, forcing the *s*^–4^ decay of the intensity at higher angles for homogeneous particles demanded by Porod’s law^53^.

The program DAMMIN^54^ was employed to construct low resolution *ab initio* bead models of PrP mRNA that best fitted the scattering data. DAMMIN employs a simulated annealing (SA) procedure to build a compact bead configuration inside a sphere with the diameter *D*_*max*_ that fits the experimental data *I*_*exp*_(*s*) to minimize the discrepancy:

2 $${\chi }^{2}=\frac{1}{N-1}{\sum }_{j}{[\frac{I({s}_{j})-c{I}_{calc}({s}_{j})}{\sigma ({s}_{j})}]}^{2}$$

where *N* is the number of experimental points, *c* is a scaling factor and *I*_calc_(*s*_*j*_) and *σ*(*s*_*j*_) are the calculated intensity and the experimental error at the momentum transfer *s*_*j*_, respectively. Fifteen independent DAMMIN runs were performed for each scattering profile in the “slow” mode, using default parameters and no symmetry assumptions (P1 symmetry). The models resulting from independent runs were superimposed using the program SUPCOMB^55^ and aligned models were averaged using DAMAVER^56^ to generate a consensus three-dimensional shape. The SAXS models of the mRNA constructs were deposited into SASDB^57^.

### Selective 2′-hydroxyl acylation analyzed by primer extension (SHAPE)

A 500 ng sample of RNA in 10 mM Tris pH7.5 buffer with 100 mM LiCl or KCl was treated with 10 mM SHAPE reagent N-methylisatoic anhydride (NMIA) or DMSO as control for 45 min at 37 °C. After precipitation, RNA was reverse transcribed with Revert Aid Minus H (Thermo Scientific) and a VIC-labeled RT-primer for 1 h at 44 °C. Subsequently, RNA was hydolysed by addition of 0.4 M NaOH and heating to 95 °C for 5 min and cDNA was separated on a denaturing 12.5% polyacrylamide gel.

### *In vivo* expression in HeLa cells

The PrP octa-repeat domain was cloned between mCherry and EYFP and EBNA1 variants were cloned C-terminally to e GFP in a pCDNA3 vector and transfected into HeLa cells (ATCC CCL-2) with polyethylenimine (PEI-40). Five to six hours post transfection the medium was replaced and 0, 2.5 or 10 µM PDS added to the cells^57^. After expression overnight, HeLa cells were analyzed by fluorescence microscopy (DMi8, Leica), subsequently harvested by trypsinization and analyzed by flow cytometry on FACS Calibur (Becton Dickinson). In addition, cells were re-suspended in SDS sample buffer, proteins separated by SDS-PAGE and blotted onto a PVDF membrane. mCherry, GFP and Neomycin phosphotransferase 2 (NPT2) as transfection control were detected by specific antibodies (St. John’s Laboratory, Roche, Milipore).

## Supplementary information

Supplementary Material
