# Author Correction: Octa-repeat domain of the mammalian prion protein mRNA forms stable A-helical hairpin structure rather than G-quadruplexes

**DOI:** 10.1038/s41598-020-61336-0

**Published:** 2020-03-04

**Authors:** Andreas Czech, Petr V. Konarev, Ingrid Goebel, Dmitri I. Svergun, Peter R. Wills, Zoya Ignatova

**Affiliations:** 1 Institute of Biochemistry and Molecular Biology University of Hamburg, Hamburg, Germany; 2 A. V. Shubnikov Institute of Crystallography of Federal Scientific Research Centre “Crystallography and Photonics” of Russian Academy of Sciences, Moscow, Russia; 3 National Research Centre “Kurchatov Institute”, Moscow, Russia; 4 European Molecular Biology Laboratory, Hamburg Outstation, c/o DESY, Hamburg, Germany; 5 Department of Physics, University of Auckland, Auckland, New Zealand

Correction to: *Scientific Reports* 10.1038/s41598-019-39213-2, published online 21 February 2019

This Article contains errors in Reference 29 which was incorrectly given as:

Wills, P. R. Editors’ forum mechanism of replication scrapie prion for Induced frameshifting an information-carrying. 235–249 (1989).

The correct reference is listed below as ref. 1:
