# Supplementary material for: Octa-repeat domain of the mammalian prion protein mRNA forms stable A-helical hairpin structure rather than G-quadruplexes
Source: Sci Rep. 2019 Feb 21;9:2465. doi: 10.1038/s41598-019-39213-2 (PMC6384910; doi:10.1038/s41598-019-39213-2)
Supplement: Supplementary file 1 — Supplementary Material [file 41598_2019_39213_MOESM1_ESM.pdf]

Octa-repeat domain of the mammalian prion protein mRNA forms stable A-helical hairpin structure rather than G-quadruplexes

Andreas Czech[1,*], Petr V. Konarev[2,3], Ingrid Goebel[1], Dmitri I. Svergun[4], Peter R. Wills[5], Zoya Ignatova[1]

[1] Institute of Biochemistry and Molecular Biology University of Hamburg, Hamburg, Germany

[2] A.V.Shubnikov Institute of Crystallography of Federal Scientific Research Centre "Crystallography and Photonics" of Russian Academy of Sciences, Moscow, Russia

[3] National Research Centre "Kurchatov Institute", Moscow, Russia

[4] European Molecular Biology Laboratory, Hamburg Outstation, c/o DESY, Hamburg, Germany

[5] Department of Physics, University of Auckland, Auckland, New Zealand

* Corresponding author: andreas.czech@chemie.uni-hamburg.de

Supplementary Table 1: Overall SAXS parameters

(a) Sample details.

	wt	wt+KCl	wt +LiCl	wt+KCl+PDS	ΔG4	ΔG4+KCl	ΔG4+LiCl	ΔG4+KCl+ PDS
Sample	wt PrP octa-repeat domain RNA				ΔG4 PrP octa-repeat domain RNA			
Organism	human				human			
Length	222 nt				222 nt			
MM from chemical composition	72410.87 Da				71810.51 Da			
Concentration range	0.25 – 2.0 µg/µl				0.25 – 2.0 µg/µl			
Preparation, purification	*in vitro* transcription (Promega RiboMax Large RNA production kit), clean up (Thermo Scientific GeneJet RNA purification kit)				*in vitro* transcription (Promega RiboMax Large RNA production kit), clean up (Thermo Scientific GeneJet RNA purification kit)			
Re-folding	5 min 95°C, min. 1 h room temperature				5 min 95°C, min. 1 h room temperature			
Solvent	10 mM Tris pH7.5				10 mM Tris pH7.5			
Additives	---	100 mM KCl	100 mM LiCl	100 mM KCl, 10 µM PDS	---	100 mM KCl	100 mM LiCl	100 mM KCl, 10 µM PDS

(b) SAXS data-collection parameters.

Instrument/data processing	P12 beamline (PETRA-III) with PILATUS 2M detector [1]
Wavelength (Å)	1.24
Beam size (mm)	0.2 x 0.12
Camera length (m)	3.000
s measurement range ($Å^{-1}$)	0.00364–0.5033
Normalization	To transmitted intensity by pin-diode counter near beam-stop
Monitoring for radiation damage	Data frame-by-frame comparison
Exposure time	Continuous 0.05 s data-x 20 frames measurements
Sample configuration	Standard SAXS measurements using the automated sample changer
Sample temperature (°C)	20

(c) Software employed for SAXS data reduction, analysis and interpretation.

SAXS data reduction	I(s) vs. s using Bequerel pipeline [2], solvent subtraction using PRIMUS (ATSAS 2.8.0; [3]
Basic analyses: Guinier, P(r), Vp	PRIMUS and GNOM from ATSAS 2.8.0 [3,4]
Shape/bead modelling	DAMMIF [5], DAMMIN [6] via ATSAS online (https://www.embl-hamburg.de/biosaxs/atsas-online/)
Validation and averaging of ab initio models	DAMAVER [7] and SUPCOMB [8]
Three-dimensional graphic model representations	MASSHA from ATSAS 2.8.0 [9]

(d) Structural parameters.

PrP mRNA	wt	wt+KCl	wt +LiCl	wt+KCl+ PDS	ΔG4	ΔG4 +KCl	ΔG4 +LiCl	ΔG4+KCl +PDS
Rg (Å)	73.2±0.7	92.5±0.9	92.4±0.9	92.9±0.9	69.3±0.7	93.9±0.9	97.8±0.9	95.9±0.9
s_{min} ($Å^{-1}$)	0.01	0.011	0.01	0.01	0.011	0.011	0.011	0.01
sRg max (s_{min} = 0.010 $Å^{-1}$)	1.3	1.3	1.3	1.3	1.3	1.3	1.3	1.3
MM from I(0)	125±22	174±33	163±32	167±31	122±24	185±33	191±31	173±32
Rg (Å)	73.5±0.5	93.0±0.7	92.8±0.7	93.2±0.7	69.6±0.5	94.3±0.7	98.0±0.7	96.0±0.7
Dmax (Å)	250±8	310±10	310±10	310±10	240±8	330±10	330±10	330±10
s range ($Å^{-1}$)	0.010-0.380	0.011-0.341	0.010-0.341	0.010-0.341	0.011-0.380	0.011-0.341	0.011-0.341	0.010-0.341
Total estimate from GNOM	0.79	0.77	0.8	0.79	0.78	0.81	0.77	0.79
M from I(0)	130±20	180±30	165±30	170±30	125±20	190±30	195±30	175±30
Porod volume (Vp) (10^3 $Å^3$)	165±20	240±30	230±30	245±30	150±20	235±30	255±30	260±30

(e) Shape model-fitting results.

PrP mRNA	wt	wt+KCl	wt +LiCl	wt+KCl+ PDS	ΔG4	ΔG4 +KCl	ΔG4 +LiCl	ΔG4+KCl +PDS
s range for fitting (Å^{-1})	0.010-0.380	0.011-0.341	0.010-0.341	0.010-0.341	0.011-0.380	0.011-0.341	0.011-0.341	0.010-0.341
Symmetry, aniso-tropy assumpt.	P1, none	P1, none	P1, none	P1, none	P1, none	P1, none	P1, none	P1, none
NSD (standard deviation)	1.17	1.18	1.12	1.14	1.14	0.84	1.15	1.11
χ^2 range	1.40-1.44	1.26-1.30	1.10-1.12	1.54-1.58	1.35-1.40	1.22-1.25	1.13-1.15	1.49-1.55
Resolution (from SASRES) (Å)	55±3	55±3	55±3	55±3	55±3	55±3	55±3	55±3
MM estimate as 0.5*volume of models (10^3 Da)	181	278	268	286	173	270	287	295

(f) SASBDB IDs for data and models.

PrP mRNA	wt	wt+KCl	wt +LiCl	wt+KCl+ PDS	ΔG4	ΔG4+KCl	ΔG4+LiCl	ΔG4+KCl +PDS
IDs	SASDDG 5	SASDDH 5	SASDD J5	SASDDK 5	SASDDL 5	SASDDM 5	SASDDN 5	SASDDP 5

Notations: R_g, radius of gyration; D_{max}, maximum size of the particle; V_p, excluded volume of the hydrated particle; MM, molecular mass; χ^2_{ab} values for the fit from *ab initio* models using DAMMIN.

1. Blanchet, C. E. *et al.* Versatile sample environments and automation for biological solution X-ray scattering experiments at the P12 beamline (PETRA III, DESY). *J. Appl. Crystallogr.* **48,** 431–443 (2015).

2. Franke, D., Kikhney, A. G. & Svergun, D. I. Automated acquisition and analysis of small angle X-ray scattering data. *Nucl. Instruments Methods Phys. Res. Sect. A Accel. Spectrometers, Detect. Assoc. Equip.* **689,** 52–59 (2012).

3. Konarev, P. V. *et al. PRIMUS* : a Windows PC-based system for small-angle scattering data analysis. *J. Appl. Crystallogr.* **36,** 1277–1282 (2003).

4. Svergun, D. I. Determination of the regularization parameter in indirect-transform methods using perceptual criteria. *J. Appl. Crystallogr.* **25,** 495–503 (1992).

5. Franke, D. & Svergun, D. I. *DAMMIF* , a program for rapid *ab-initio* shape determination in small-angle scattering. *J. Appl. Crystallogr.* **42,** 342–346 (2009).

6. Svergun, D. I. Restoring Low Resolution Structure of Biological Macromolecules from Solution Scattering Using Simulated Annealing. *Biophys. J.* **76,** 2879–2886 (1999).

7. Volkov, V. V. & Svergun, D. I. Uniqueness of *ab initio* shape determination in small-angle scattering. *J. Appl. Crystallogr.* **36,** 860–864 (2003).

8. Kozin, M. B. & Svergun, D. I. Automated matching of high- and low-resolution structural models. *J. Appl. Crystallogr.* **34,** 33–41 (2001).

9. Konarev, P. V., Petoukhov, M. V & Svergun, D. I. MASSHA – a graphics system for rigid-body modelling of macromolecular complexes against solution scattering data MASSHA ± a graphics system for rigid-body modelling of macromolecular complexes against solution scattering data. *J. Appl. Cryst* **34,** 527–532 (2001).

10. Wei, J. *et al.* Ubiquitous Autofragmentation of Fluorescent Proteins Creates Abundant Defective Ribosomal Products (DRiPs) for Immunosurveillance. *J. Biol. Chem.* **290,** 16431–9 (2015).

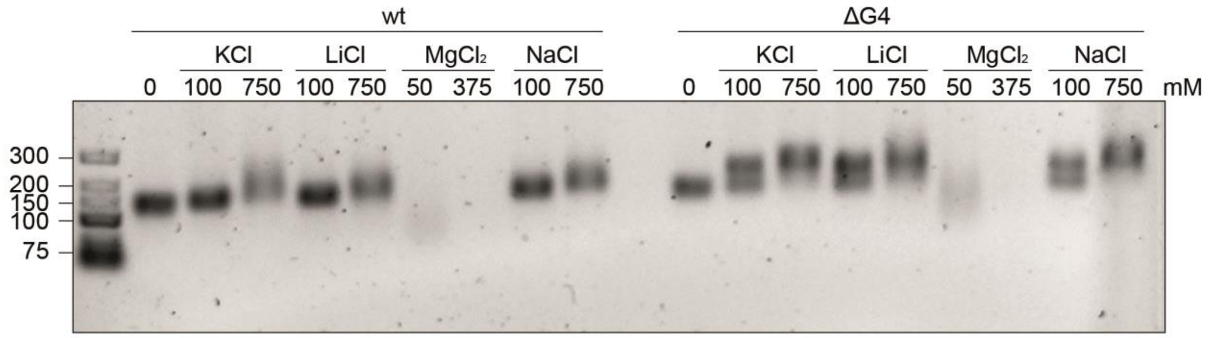

Supplementary Fig. 1. Native agarose gel electrophoresis of wt and ΔG4 PrP octa-repeat mRNA.

Different salts promote oligomerization of wt and ΔG4 PrP octa-repeat mRNA. $MgCl_2$ addition causes fragmentation of RNA.

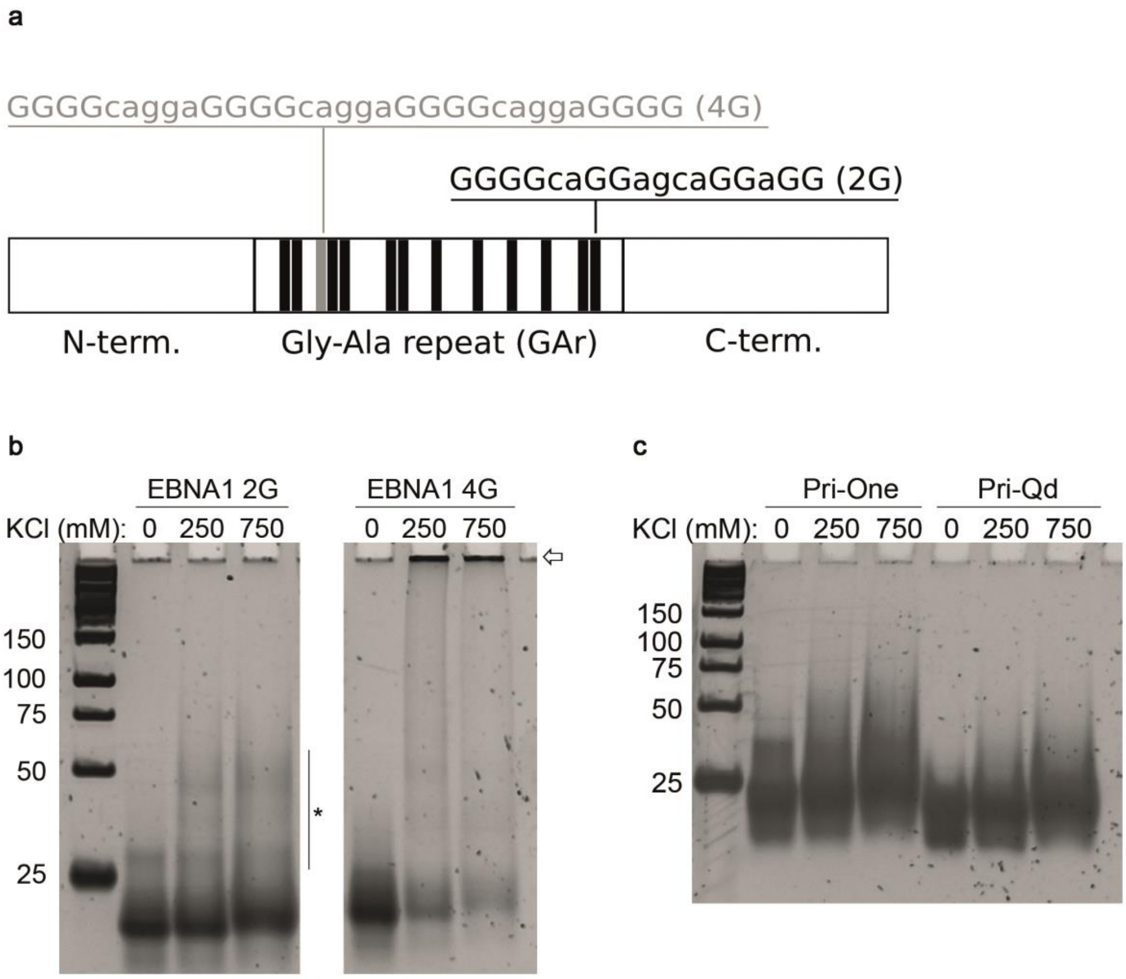

Supplementary Fig. 2. Native polyacrylamide gel electrophoresis of known G-quadruplexes.

a. Scheme of EBNA1 mRNA bearing 12 two-layer (2G) and one four-layer (4G) G-quadruplexes in the glycine-alanine repeat domain (GAr). **b.** Isolated 2G and 4G G-quadruplexes form dimers and trimers (asterix) and high molecular weight oligomers (arrow), respectively, upon incubation with KCl. **c.** Isolated putative G-quadruplexes Pri-One and Pri-Qd might form dimers, but no high-molecular weight oligomers.

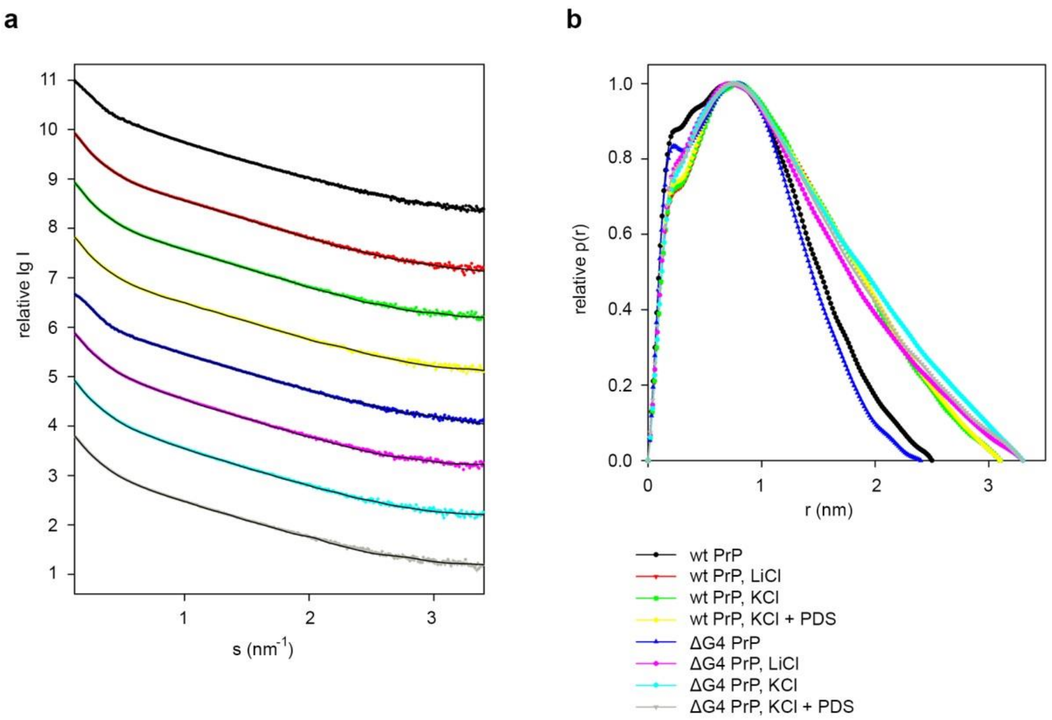

Supplementary Fig. 3. Experimental X-ray scattering pattern and distance distribution functions of PrP mRNA.

a. Scattering profiles in solution. Dots with error bars denote the experimental scattering data. The fits obtained by DAMMIN are displayed as solid lines. The plot displays the logarithm of the scattering intensity as a function of momentum transfer. The curves are displaced along the vertical logarithmic axis by one logarithmic order for clarity. **b.** Distance distribution functions of PrP octa-repeat mRNA computed from the experimental X-ray scattering patterns using GNOM and normalized to a maximum value of unity.

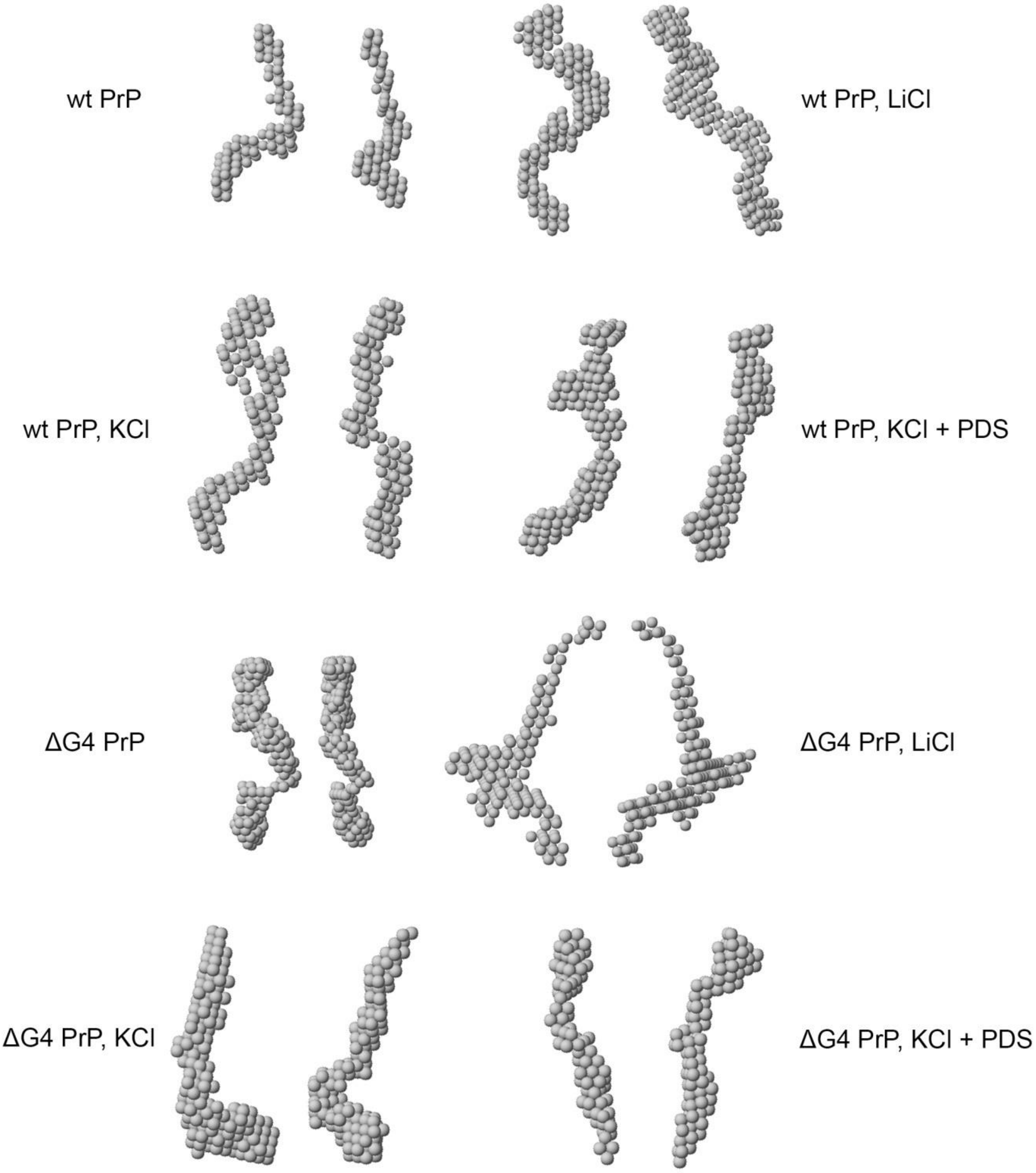

Supplementary Fig. 4. Gallery of averaged filtered *ab initio* models of PrP mRNA.

Right views are rotated counterclockwise by 90 ° around the y-axis.

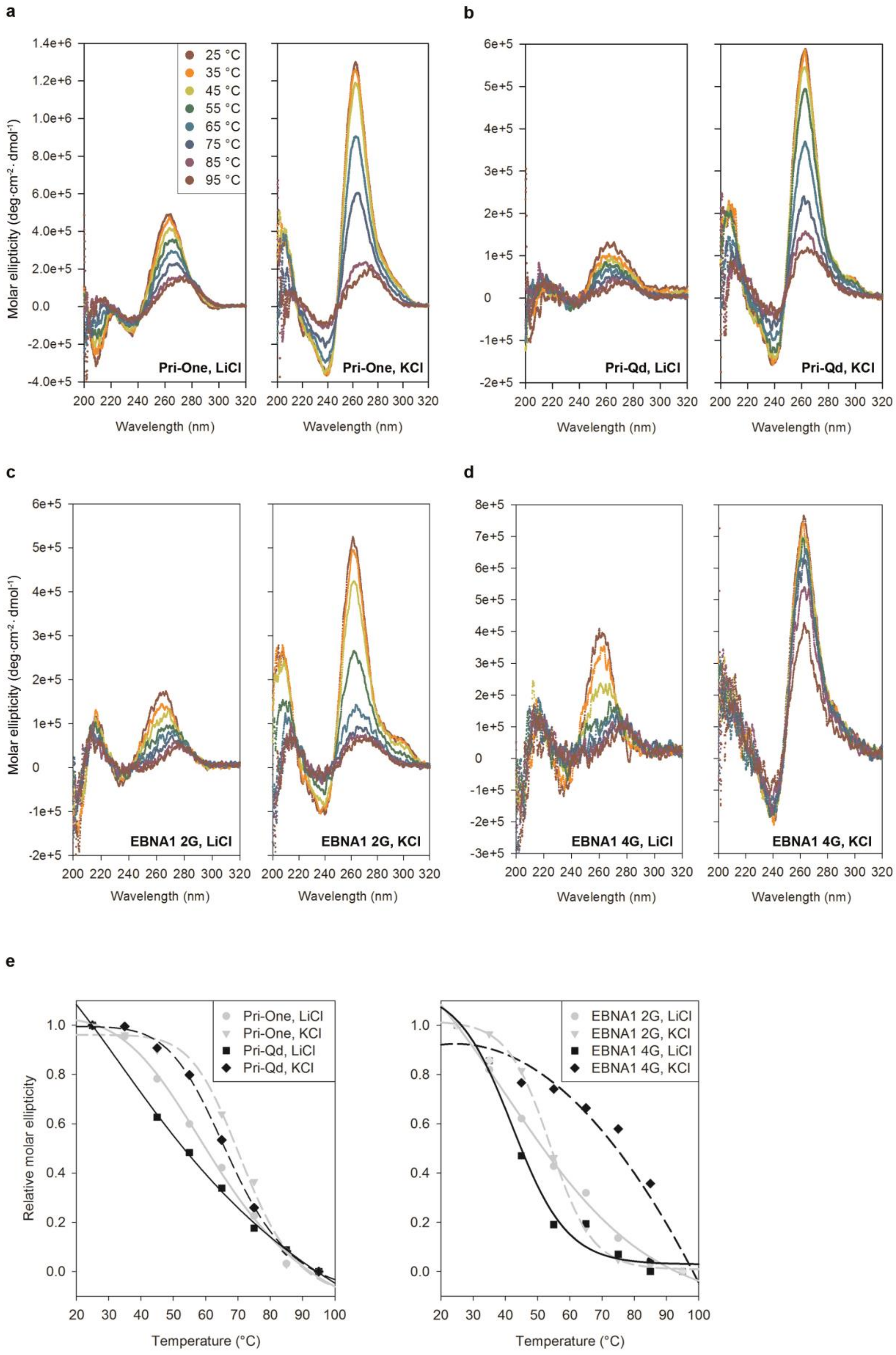
a
b
c
d
e
25 °C
35 °C
45 °C
55 °C
65 °C
75 °C
85 °C
95 °C
Molar ellipticity (deg·cm⁻²·dmol⁻¹)
Wavelength (nm)
Pri-One, LiCl
Pri-One, KCl
Pri-Qd, LiCl
Pri-Qd, KCl
EBNA1 2G, LiCl
EBNA1 2G, KCl
EBNA1 4G, LiCl
EBNA1 4G, KCl
Relative molar ellipticity
Temperature (°C)

Supplementary Fig. 5. CD spectroscopy of known G-quadruplexes.

CD spectra of isolated G-quadruplex stretches from PrP, **a.** Pri-One and **b.** Pri-Qd, and from EBNA1, **c.** 2G and **d.** 4G, at temperatures ranging from 25 to 95 °C in the presence of LiCl or KCl. **e.** Melting curves extracted from maximal ellipticity and normalized to the molar ellipticity at 25 °C. Points were fitted to a 4 parameter sigmoidal curve, except EBNA1 4G, KCl, which was fitted to quadratic function.

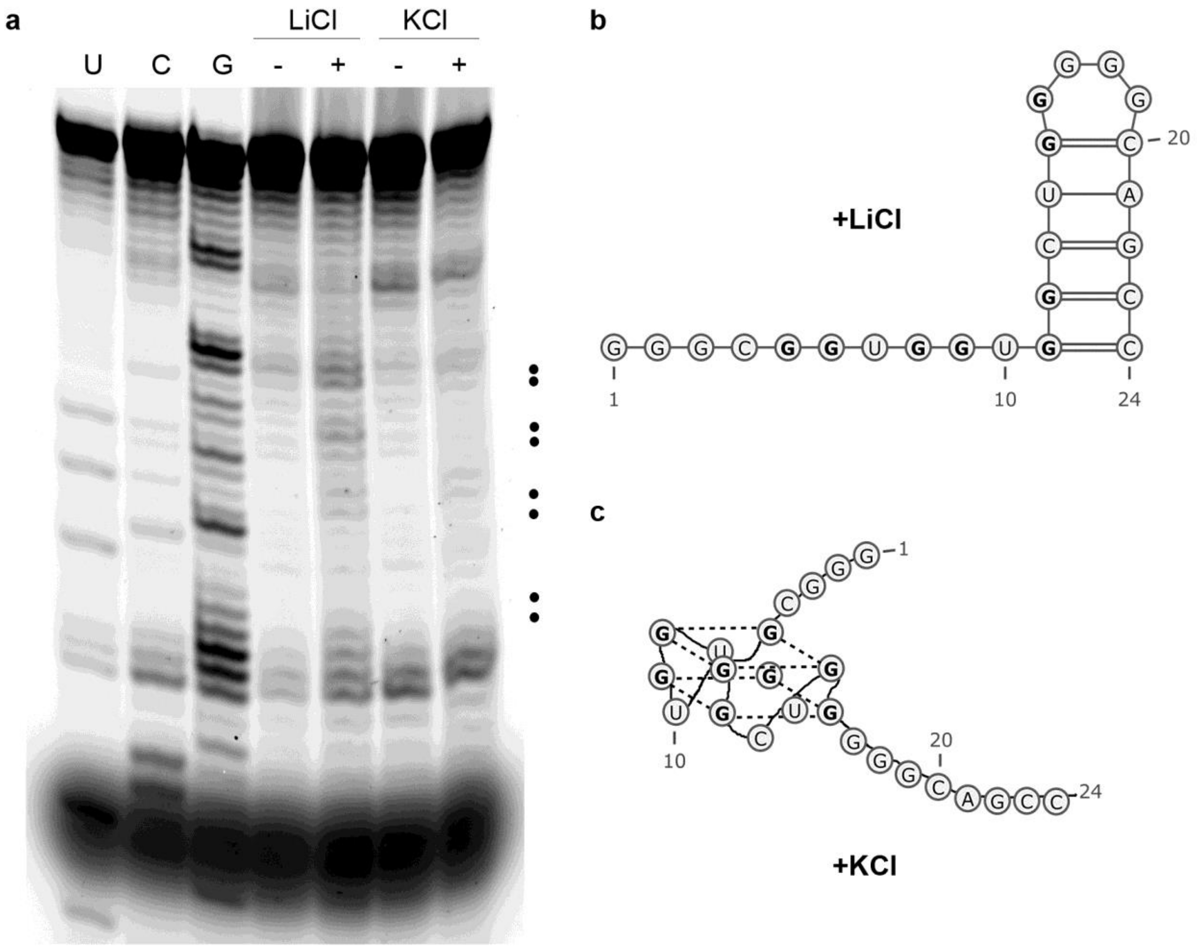

Supplementary Fig. 6. SHAPE analysis of isolated PrP stretch Pri-one.

a. SHAPE analysis of Pri-One RNA in the presence 100 mM LiCl or KCl without (–) or with SHAPE reagent NMIA (+). The RNA in the negative control (-) was incubated with DMSO. Black dots mark guanine residues involved in G-quadruplex formation. **b.** RNA secondary structure of Pri-One forming hairpin in the presence of LiCl. **c.** RNA secondary structure of Pri-One forming G-quadruplex in the presence of KCl.

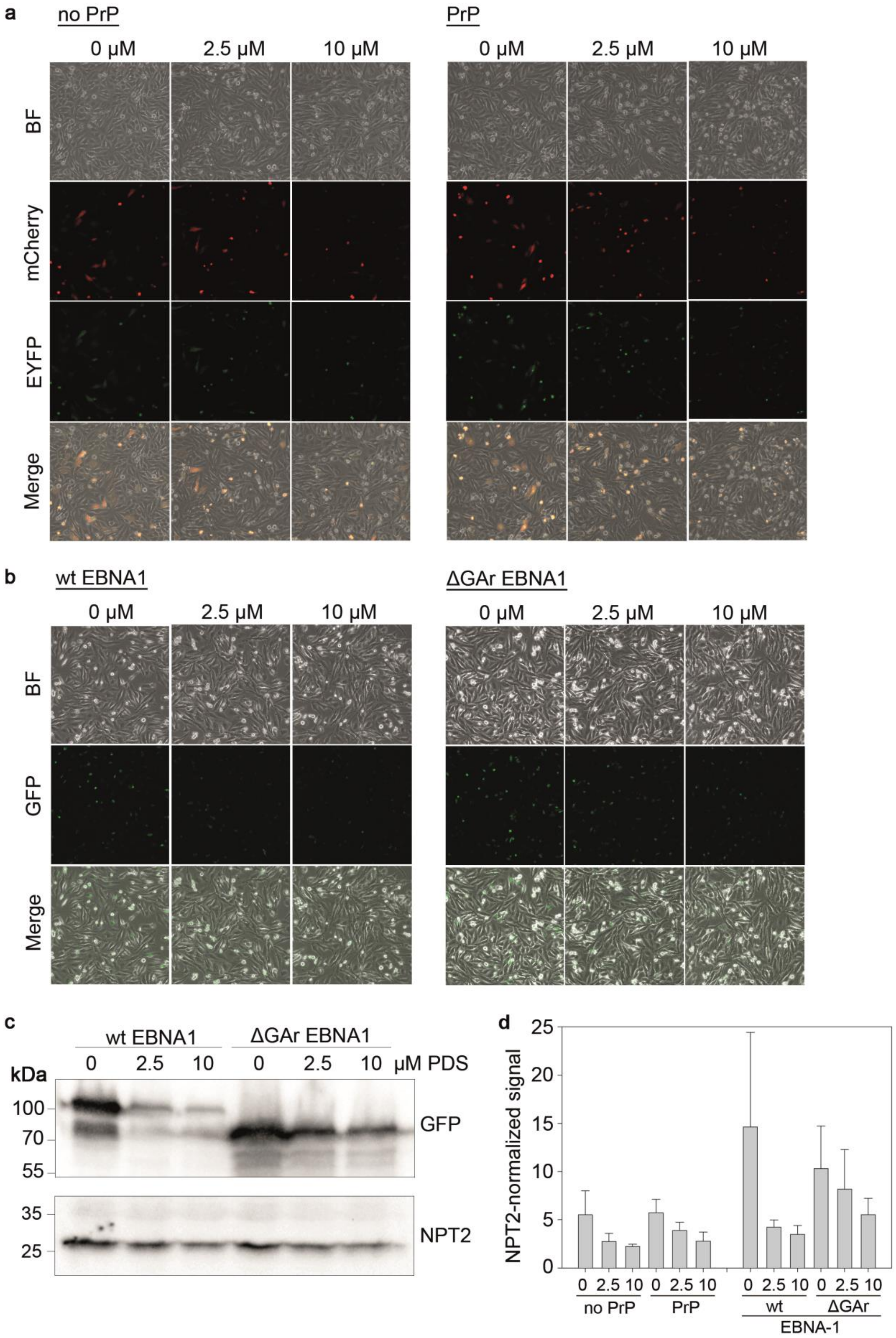
a
no PrP
PrP
0 µM
2.5 µM
10 µM
BF
mCherry
EYFP
Merge
b
wt EBNA1
ΔGAr EBNA1
GFP
c
wt EBNA1
ΔGAr EBNA1
0 2.5 10 0 2.5 10 µM PDS
kDa
100
70
55
35
25
GFP
NPT2
d
NPT2-normalized signal
25
20
15
10
5
0
0 2.5 10 0 2.5 10 0 2.5 10 0 2.5 10
no PrP
PrP
wt
ΔGAr
EBNA-1

Supplementary Fig. 7. Impact of G-quadruplex formation on protein expression *in vivo*.

a. Fluorescence microscopy of double-reporter constructs with and without PrP insert (Fig.5A) in HeLa cells in the presence of increasing PDS concentrations. **b.** Fluorescence microscopy of GFP-labeled full-length wildtype EBNA1 and a variant without the G-quadruplex-bearing glycine-alanine repeat domain (GAr). **c.** Representative immunoblot (n = 3) of EBNA1 variants detected by anti-GFP antibody. NPT2 served as transfection control. **d.** For each PDS concentration immunoblots for no PrP, PrP, wt EBNA1 or dGAr EBNA1 and the respective NPT2 band were quantified (n = 3). For normalization, signals of PrP, no PrP, wt EBNA1 or dGAr EBNA1 were divided by the respective NPT2 signal of the same sample. NPT2-normalized signals at 0, 2.5 and 10 µM PDS are plotted as means ± SD.

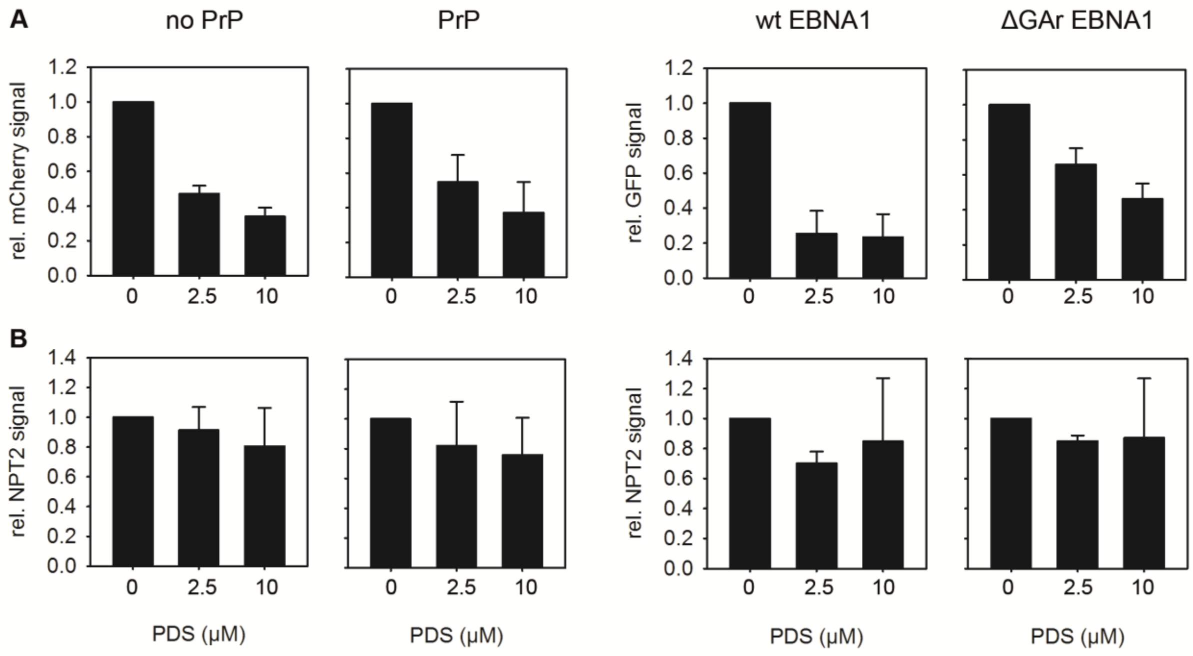

Supplementary Fig. 8. Impact of Pyridostatin on protein expression

Immunoblots of no PrP, PrP, wt EBNA1 and ΔGAr EBNA1 as well as respective NPT2 bands from cells treated with 0 – 10 µM PDS were quantified and normalized by dividing by the respective signal at 0 µM PDS. Data are means ± SD (*n* = 3).

Supplementary Table 2: QGRS mapper prediction of number and probability (G-score) of potential G-quadruplexes within studied sequences

Sequence	Length	No. of G4	No. of G4 per 100nt	G-score range	G-score average
no PrP (mCherry-EYFP)	1179 nt	16	1.36	9 – 33	17.1
ΔGAr EBNA1	1194 nt	16	1.34	7 – 21	18.6
NPT2	795 nt	8	1.01	11 – 20	14.6
GAr	447 nt	17	3.80	18 – 42	21.9
PrP octa-repeat domain	195 nt	6	3.08	15 – 21	19.5

Kikin, O., D’Antonio, L. & Bagga, P. S. QGRS Mapper: A web-based server for predicting G-quadruplexes in nucleotide sequences. *Nucleic Acids Res.* **34,** 676–682 (2006).

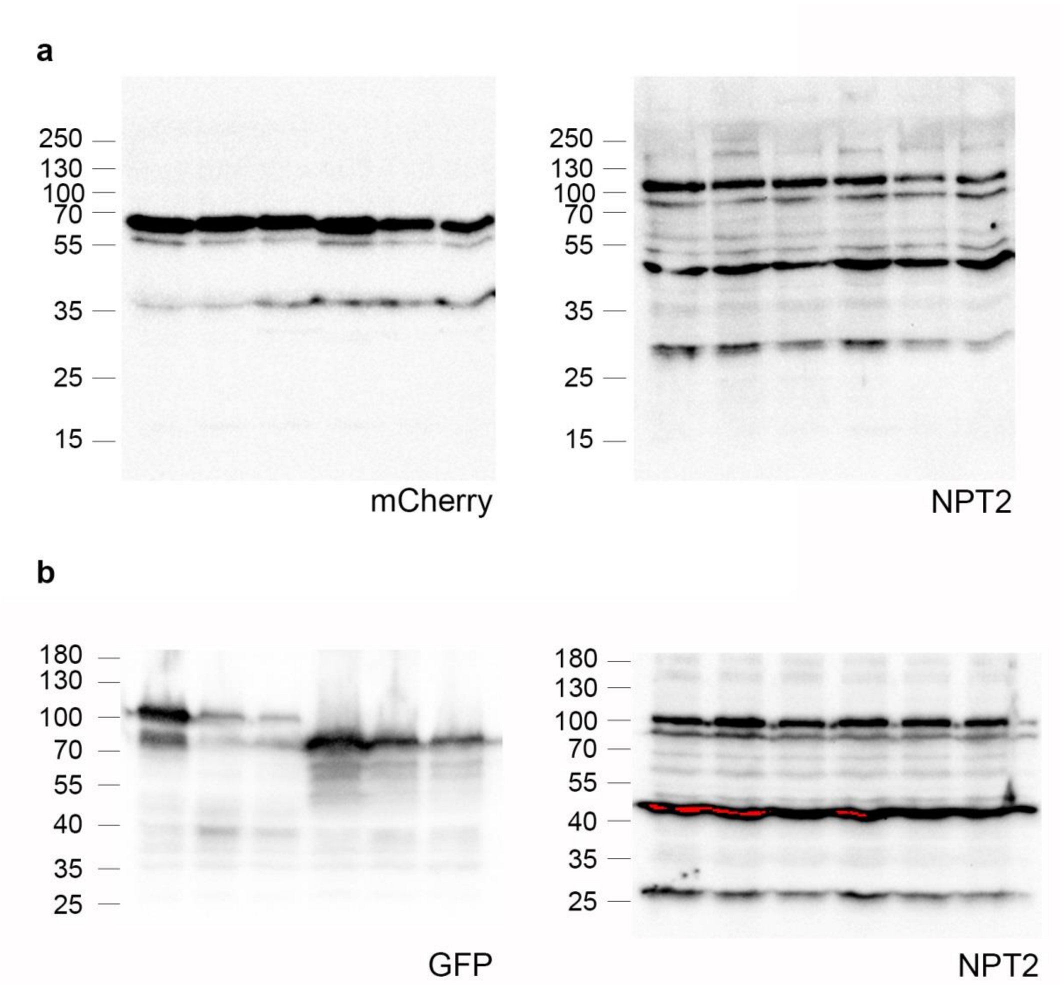

Supplementary Fig. 9. Full size immunoblots (basis for Fig. 5C and Supplementary Fig. 7c)
